# A controlled survey of less typical long-term consequences after an extensive waterborne epidemic

**DOI:** 10.1186/s12879-017-2260-9

**Published:** 2017-02-21

**Authors:** Janne Laine, Outi Laine, Jukka Lumio, Jaakko Antonen, Salla Toikkanen, Mikko J. Virtanen, Markku Kuusi, the Pirkanmaa Waterborne Outbreak Study Group, Pekka Collin, Jaakko Herrala, Tina Katto, Markku Korpela, Eila Kujansuu, Anna-Leena Kuusela, Sami Mustajoki, Jukka Mustonen, Heikki Oksa, Petri Ruutu, Sirpa Räsänen, Terhi Uotila

**Affiliations:** 1 Department of Internal Medicine, Tampere University Hospital, P.O. Box 2000, 33521 Tampere, Finland; 2 National Institute of Health and Welfare, Helsinki, Finland; 3 School of Medicine, University of Tampere, Tampere, Finland

**Keywords:** Waterborne infections, Epidemics, Gastroenteritis, General symptoms, Outcome

## Abstract

**Background:**

Extensive backflow of treated wastewater caused household water contamination in a Finnish town in 2007. The drinking water of 9 500 residents became heavily polluted with faecal microbes, resulting in a large gastroenteritis epidemic. Cases of reactive arthritis, milder joint symptoms and prolonged gastrointestinal symptoms were observed after the outbreak. A follow-up survey was performed to study less familiar long-term health consequences within a year from the outbreak.

**Methods:**

The contaminated group comprised a sample of residents of the area with polluted water supply (*N* = 323) and the control group a sample of residents in a nearby municipality (*N* = 186). The presence of 20 general symptoms or complaints was inquired by a mail survey. Quarterly prevalence of each symptom or complaint was measured. Twelve of these proceeded to further analysis.

**Results:**

The response rate was 53% (323/615) in the contaminated group and 54% (186/343) in the control group. Rash, eye irritation, heartburn and weight loss were more prevalent in the contaminated group during the first year quarter. In the last year quarter, only eye irritation was significantly more common in the contaminated group.

**Conclusion:**

The excess prevalence of four complaints at the first year quarter can be explained by acute gastroenteritis or intensive water chlorination. The excess prevalence of eye irritation at the fourth year quarter cannot be explained by chlorination anymore but might be a sign of co-existing reactive joint disease. In general, long-term consequences of the outbreak can be considered minor in terms of the surveyed symptoms or complaints.

## Background

Outbreaks associated with drinking water remain an important concern for public health, even in countries with well-maintained water supply and sewerage systems. Several epidemics with substantial morbidity due to contaminated household water have been described. In some occasions, these epidemics have caused mortality [1–3].

In addition to immediate health effects, a waterborne disease may cause late effects. Acute gastroenteritis is usually the initial disease associated with waterborne epidemics. Reactive arthritis (ReA), milder forms of joint complaints and prolonged gastrointestinal symptoms are well-known consequences of acute gastroenteritis. Studies concerning late effects of these outbreaks have therefore mainly focused on these complications [4–8]. However, as a large waterborne epidemic is a major incident affecting considerably both individual health and public health, other long-term health consequences may also occur. The presence of these other consequences has not been widely studied. Increased incidence of hypertension, cardiovascular disease and chronic fatigue syndrome have been reported to be associated with exposure to waterborne pathogens in two major waterborne epidemics [7, 9].

A large drinking water–associated gastroenteritis epidemic was noticed at the end of November 2007 in a Finnish town of Nokia (population 30 000) [10]. A valve connecting wastewater effluent and household water distribution lines was opened during maintenance work at the town’s wastewater plant and, by accident, left open for 2 days. A large amount of treated wastewater flowed through this cross-connection to household water distribution line. The drinking water of 9 500 residents became severely contaminated with faecal microbes, and as a result several thousand residents fell ill with gastroenteritis.

A comprehensive epidemiological investigation was launched to study the immediate disease burden, health-economic costs and delayed health effects caused by the epidemic [10–13]. Altogether 53% of the residents in the area of contaminated water reported illness and excess morbidity was observed also in the uncontaminated part of the town. Stool samples revealed seven pathogens, most importantly *Campylobacter spp.,* norovirus and *Giardia spp.* All organisms found from patient samples were detected also from water and/or network samples [10]. Altogether 148 cases of campylobacteriosis and 55 cases of giardiasis were laboratory-confirmed. Only few stool samples from patients were investigated for norovirus. However, as all these specimens were positive, norovirus was detected in water samples and there were signs of person-to-person transmission, norovirus was considered to be a main pathogen as well.

Altogether 21 cases of ReA were confirmed and a third of them were still on antirheumatic medication 1 year after [14, 15]. Of those who had fallen ill with gastroenteritis during the epidemic, 19% still had joint symptoms and 11% gastrointestinal symptoms at 15 months from the epidemic [12].

We aimed to evaluate the less frequently studied health effects of a waterborne epidemic during the following 12 months.

## Methods

Two questionnaire studies were conducted, the first at 8 weeks and the second (a follow-up study) at 15 months from the incident. Both studies were carried out by using paper-and-pencil mail surveys. A reminder letter was sent to non-responders of the first study after 3 weeks but not to non-responders of the follow-up study. The present report utilizes the data from the follow-up study.

Three areas were defined: the contaminated and uncontaminated parts of the town constituted two areas. These areas were determined by using microbiological data from water samples and technical modelling of water-flow directions within the pipeline network. A municipality on the opposite side of the urban area served as a control area. Three study groups were then created by randomly sampling 1 000 residents from each of these areas: contaminated, uncontaminated and control groups [10]. The participants were obtained from the national population register with geographical coordinates of their residence, enabling reliable division between contaminated and uncontaminated groups. Participants of all ages were included and groups were matched with age and gender. Only one study participant per household was permitted.

The follow-up study was based on the same study groups; however, only those responding to the first survey and giving their permission to be re-contacted were included. Therefore the study groups in the follow-up study became a subset of the original sample (Table 1) [12]. The participants were asked about general symptoms or complaints occurring within 12 months (from 1 January to 31 December 2008) after the epidemic. We could not identify a validated set of questions concerning general physical symptoms. We therefore reviewed several combinations of questions used in primary care and occupational health care and formulated a combination of 20 symptoms or complaints (Table 2). The presence of these was asked month by month. To make the analysis straightforward, three subsequent months were pooled to constitute a quarter of the year (1q–4q). The quarterly prevalence of the asked symptoms was then counted. Because epidemic-related morbidity was observed also in the uncontaminated group in the first survey, it was considered to be a biased control group and excluded from this study.

**Table 1:** Evolution of the study groups in the follow-up study (adapted from reference 12)

	Contaminated group	Control group
Population 2007	9 538	27 259
Original sample size	1 021^a^	1 000
Responded to the first study *N* (%)	808 (79%)	598 (60%)
Sample size in the follow-up study (i.e., those who gave permission to be contacted again)	615	343
Responded to the follow-up study *N* (%) *% of the original sample*	323 (53%) *32%*	186 (54%) *19%*
Having had epidemic-related gastroenteritis during the epidemic, according to the follow-up study	174 (54%)	6 (3%)

^a^The original sample size was 1000 persons in all groups. Assessment of the contaminated area became more precise later, and 21 participants were shifted to the contaminated group

**Table 2:** Twenty symptoms or complaints that were originally asked in the follow-up study

**Headache**	
**Dizziness**	
Memory disturbance	
**Anxiety or fear**	
Chest pain	
**Fatigue**	
**Rash**	
**Dyspnoea**	
Cough	
**Eye irritation**	
**Heartburn**	
Diarrhea	
**Dysuria**	
Urinary tract infection	
Neck pain	
**Back pain**	
Weight gain	
**Weight loss**	
**Sleeping disturbances**	
Difficulty in sexual performance	

Twelve of symptoms or complaints (bolded) proceeded to further analysis

The evolution of the study groups from the original sample to the follow-up study is presented in Table 1. The analysis of selection in the follow-up study has been published before in a paper presenting the duration of gastrointestinal and joint symptoms [12]. The response rates for the follow-up were 53% (323/615) in the contaminated group and 54% (186/343) in the control group. The odds ratios (OR) for being included in the follow-up study were 1.2 (95% CI 1.0–1.5) for females, 1.7 (1.3–2.1) for those who had gastroenteritis during the epidemic and 1.7 (1.3–2.2) for those who had early joint symptoms. For responding to follow-up study, the figures were 1.1 (0.9–1.1), 1.0 (0.8–1.2) and 1.03 (0.8–1.3), respectively. 54% (174/323) of respondents in the contaminated group and 3% (6/186) in the control group stated having had gastroenteritis during the time of the epidemic.

### Statistical methods

The results are presented as prevalences. Fisher’s exact test was used to test the differences between the groups for each time point. The change in prevalence was analyzed by calculating the ratio of having a symptom at 1q–not having at 4q/not having at 1q– having at 4q (yes – no/no-yes). These ratios were analysed across the study groups for testing the statistical significance, again using Fisher’s exact test. All analyses were done with R version 3.1.

## Results

After a preliminary analysis of the data, evaluation was restricted to 12 most prevalent symptoms or complaints reported: headache, dizziness, anxiety or fear, fatigue, rash, dyspnoea, eye irritation, heartburn, dysuria, back pain, weight loss and sleeping disturbances. Seven symptoms or complaints were omitted because only few or none participants reported having them. The omitted symptoms or complaints were: memory disturbances, chest pain, rhinitis or cough, urinary tract infection, neck or shoulder pain, weight gain, sexual difficulties. Diarrhea was rejected because the duration of gastrointestinal symptoms was already evaluated in the previous study [12].

The prevalence of symptoms or complaints in the first and last year quarters is presented in Table 3. Rash, eye irritation, heartburn and weight loss were significantly more prevalent in the contaminated group in comparison to the control group in the first year quarter. In the last quarter, no excess prevalence was noted for the three first mentioned symptoms but eye irritation remained significantly more prevalent in the contaminated group. Dyspnoea was more common in the contaminated group in the last quarter but not in the first quarter.

**Table 3:** Prevalence (*N* (%)) of general symptoms in study groups in the first and last year quarters

		Contaminated group	Control group	*p*-value
		*N* = 323	*N* = 186	
Headache	1q	54 (16.7)	23 (12.2)	
	4q	47 (14.5)	36 (19.2)	
Dizziness	1q	20 (6.2)	5 (2.7)	
	4q	12 (3.7)	6 (3.2)	
Anxiety or fear	1q	11 (3.4)	6 (3.2)	
	4q	7 (2.2)	14 (7.5)	
Fatigue	1q	58 (17.9)	22 (11.7)	
	4q	29 (9.0)	34 (18.1)	0.03
Rash	1q	38 (11.7)	10 (5.3)	0.02
	4q	23 (7.1)	16 (8.5)	
Dyspnoea	1q	14 (4.3)	3 (1.6)	
	4q	15 (4.6)	2 (1.1)	0.04
Eye irritation	1q	24 (7.4)	5 (2.7)	0.03
	4q	17 (5.3)	2 (1.1)	0.02
Heartburn	1q	27 (8.3)	6 (3.2)	0.02
	4q	23 (7.1)	12 (6.4)	
Dysuria	1q	8 (2.5)	2 (1.1)	
	4q	9 (2.8)	3 (1.6)	
Back pain	1q	35 (10.8)	13 (6.9)	
	4q	34 (10.5)	22 (11.7)	
Weight loss	1q	26 (8.0)	4 (2.1)	0.01
	4q	8 (2.5)	2 (1.1)	
Sleeping disturb.	1q	36 (11.1)	11 (5.9)	
	4q	24 (7.4)	15 (8.0)	

Only *p*-values indicating statistical significance are shown

1q first quarter, 4q\ fourth quarter

**Fig. 1:**
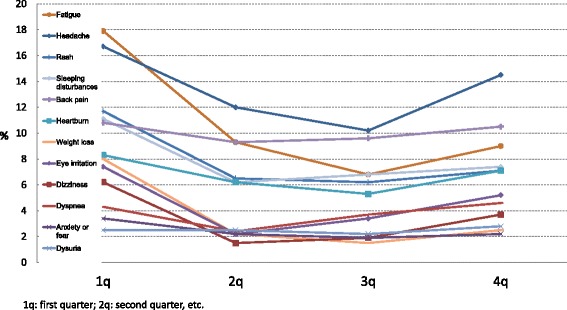
Prevalence (%) of general symptoms in the contaminated group by quarter of the year

Quarterly changes in prevalence of various symptoms or complaints in the contaminated group are presented in Fig. 1. The prevalence of all these symptoms decreased from the first quarter to the second, but thereafter some increase took place. The decline in prevalence of headache, anxiety or fear, fatigue, rash, heartburn, back pain and sleeping disturbances from the first quarter to the fourth quarter was statistically significant.

## Discussion

The epidemic in Nokia was the most significant waterborne epidemic in Finland to date. The well-known consequences of bacterial gastroenteritis, i.e. reactive arthritis and sustained gastrointestinal complaints, were both observed after the Nokia epidemic [12–15].

The occurrence of long-term consequences other than gastrointestinal or joint complaints after waterborne outbreaks has not been extensively studied. We now evaluated the prevalence of 12 general symptoms or complaints over a period of 12 months. These symptoms were more commonly observed in the contaminated group than in the control group during the first year quarter, but only rash, eye irritation, heartburn and weight loss reached the level of statistical significance. However, only eye irritation remained more prevalent in the contaminated group 1 year after the outbreak.

Most of the decrease took place from the first year quarter to the second. Seasonal variation may explain the moderate increase noted from the third quarter to the fourth, as the last year quarter was late autumn with a higher seasonal incidence of common cold and viral gastroenteritis. This may also explain the excess prevalence of dyspnoea in the contaminated group in the last quarter. The contaminated part of the town is an area with lots of families with children. Small children have a high incidence of respiratory tract infections during cold season, which may cause dyspnoea. However, in the pre-analysis, there were very few cases of rhinitis or cough. Nevertheless, as there was no difference between the prevalence of dyspnoea in the first and last quarters within the contaminated group, we conclude that the difference against the control group in the last quarter was not due to the epidemic.

Weight loss and heartburn are plausible signs of acute gastroenteritis, and the higher prevalence of these symptoms observed in the contaminated group was likely due to the acute disease. There was no excess of these symptoms left at the end of the study period, indicating that they did not become long-lasting conditions. Our previous study demonstrated that symptoms suggestive of irritable bowel syndrome in the contaminated group declined to the average level of general population in fifteen months [12]. Therefore, the findings of these two studies concerning the Nokia epidemic support each other. Our observations are not in line with the findings of the outbreak in Walkerton, Canada, where dyspepsia was still present 8 years after the incident [16]. However, a shiga-toxin producing *Escherichia Coli* O157:H7 was common in Walkerton. The differences in the causative pathogens may play an important role in the long-term consequences of an outbreak and therefore, it is not straightforward to compare one epidemic to another.

Rash and eye irritation were found to be more common in the contaminated group than among controls in the first quarter. Household water was intensively chlorinated for almost 3 months (i.e. almost the whole first year quarter) in the contaminated area and this could explain the excess presence of these symptoms during the first year quarter. However, eye irritation was still more prevalent in the contaminated group in the last year quarter, several months after the vigorous disinfection processes had ceased. Conjunctivitis is a co-existing sign of ReA and, therefore, this observation may be linked to the presence of reactive joint disease in the exposed population [17]. There were only 21 verified cases of ReA after this epidemic; however, milder forms of joint symptoms were common in the contaminated area, and a substantial proportion of these symptoms was still present after 15 months [12–14]. In a 1-year follow-up study of two waterborne *Cryptosporidium hominis* outbreaks in Sweden, a parallel observation was made: frequency of ocular pain was slightly increased among cases [8]. However, the difference in that study was not statistically significant.

*Giardia* was one of the pathogens detected in the Nokia epidemic, and 55 cases were verified by stool examination [18]. As the researchers of the Bergen *Giardia*-outbreak in Norway observed a 46% frequency of chronic fatigue 3 years after the waterborne epidemic, chronic fatigue may have been present after the Nokia epidemic as well. However, the prevalence of fatigue was not found to be significantly higher in the contaminated group in our study. Fatigue was instead more frequent in the control group at the end of the observation period, a finding that is difficult to explain. Because the contaminated group in the follow-up survey represented only 3.4% of the whole population in the contaminated area, probably only few cases of giardiasis were included in the follow-up study. Therefore, our results cannot challenge the findings of the Bergen study.

There are potential limitations of this study. Although the original study sample was carefully created and reflected well the underlying population, the step-wise evolution of the sample in the follow-up study may have caused bias (i.e. responding to the first survey, giving permission to be re-contacted and finally responding to the second survey). Moderate selection was detected in giving permission; those who fell ill with gastroenteritis or had joint symptoms were more prone to be included in the follow-up survey [12]. This bias was statistically significant, but not substantial. No additional differences were observed among the respondents of the follow-up study. The 15 months’ interval from the epidemic to the time of the follow-up survey may have created a recall-bias of some degree. As the symptoms or complaints asked were common ones, remembering the exact presence and timing may have been difficult. This probably holds true especially for the control group. Finally, as this was a questionnaire study, the results should be interpreted with some caution. The self-reported symptoms reflect the participants’ subjective experience and therefore, the data collected may not be completely precise.

## Conclusions

Only few general symptoms or complaints were observed in excess degree within the year after the extensive waterborne epidemic. The excess prevalence of rash, heartburn and weight loss during the first year quarter can all be explained with acute gastroenteritis or water disinfection procedures. Only the excess prevalence of eye irritation persisted throughout the follow-up time. This may be a sign of co-existing reactive joint disease. The findings support the previous impression of a relatively favorable outcome of this epidemic [10, 12, 13]. The contamination itself was a potentially dangerous event and if the pathogens involved had been more virulent, the burden of illness in this epidemic might have been substantially higher.

## Abbreviations

1q–4q: 1–4 year quarters
OR: Odds ratio
ReA: Reactive arthritis
